# Response to Early Generation Genomic Selection for Yield in Wheat

**DOI:** 10.3389/fpls.2021.718611

**Published:** 2022-01-11

**Authors:** David Bonnett, Yongle Li, Jose Crossa, Susanne Dreisigacker, Bhoja Basnet, Paulino Pérez-Rodríguez, G. Alvarado, J. L. Jannink, Jesse Poland, Mark Sorrells

**Affiliations:** ^1^International Maize and Wheat Improvement Center, Texcoco, Mexico; ^2^BASF Wheat Breeding, Sabin, MN, United States; ^3^School of Agriculture, Food and Wine, Faculty of Sciences, The University of Adelaide, Adelaide, SA, Australia; ^4^Colegio de Postgraduados, Texcoco, Mexico; ^5^USDA-ARS, Robert W. Holley Center for Agriculture and Health, Ithaca, NY, United States; ^6^Plant Breeding and Genetics Section, School of Integrative Plant Science, Cornell University, Ithaca, NY, United States; ^7^Department of Plant Pathology, Kansas State University, Manhattan, KS, United States

**Keywords:** early generation genomic selection, linear and non-linear kernels genomic matrices, wheat breeding, breeding methodology, response to selection

## Abstract

We investigated increasing genetic gain for grain yield using early generation genomic selection (GS). A training set of 1,334 elite wheat breeding lines tested over three field seasons was used to generate Genomic Estimated Breeding Values (GEBVs) for grain yield under irrigated conditions applying markers and three different prediction methods: (1) Genomic Best Linear Unbiased Predictor (GBLUP), (2) GBLUP with the imputation of missing genotypic data by Ridge Regression BLUP (rrGBLUP_imp), and (3) Reproducing Kernel Hilbert Space (RKHS) a.k.a. Gaussian Kernel (GK). F2 GEBVs were generated for 1,924 individuals from 38 biparental cross populations between 21 parents selected from the training set. Results showed that F2 GEBVs from the different methods were not correlated. Experiment 1 consisted of selecting F2s with the highest average GEBVs and advancing them to form genomically selected bulks and make intercross populations aiming to combine favorable alleles for yield. F4:6 lines were derived from genomically selected bulks, intercrosses, and conventional breeding methods with similar numbers from each. Results of field-testing for Experiment 1 did not find any difference in yield with genomic compared to conventional selection. Experiment 2 compared the predictive ability of the different GEBV calculation methods in F2 using a set of single plant-derived F2:4 lines from randomly selected F2 plants. Grain yield results from Experiment 2 showed a significant positive correlation between observed yields of F2:4 lines and predicted yield GEBVs of F2 single plants from GK (the predictive ability of 0.248, *P* < 0.001) and GBLUP (0.195, *P* < 0.01) but no correlation with rrGBLUP_imp. Results demonstrate the potential for the application of GS in early generations of wheat breeding and the importance of using the appropriate statistical model for GEBV calculation, which may not be the same as the best model for inbreds.

## Introduction

Genomic selection (GS) (Meuwissen et al., 2001; Bernardo and Yu, 2007) has become possible through the rapid development of next-generation sequencing technologies that allow the use of abundant and low-cost molecular markers. Evidence in plant breeding literature has shown that GS provides an important increase in prediction accuracy compared to pedigree and marker-assisted selection for low heritability traits (de los Campos et al., 2009, 2010, 2013; Crossa et al., 2010, 2011, 2013, 2014; González-Camacho et al., 2012, 2016; Heslot et al., 2012, 2014; Hickey and Gorjanc, 2012; Pérez-Rodríguez et al., 2012; Riedelsheimer et al., 2012; Windhausen et al., 2012; Zhao et al., 2012). An initial review of the main activities of GS in the International Maize and Wheat Improvement Center (CIMMYT) maize and wheat breeding programs was published by Crossa et al. (2014). Simultaneously, breeding programs around the world have been studying GS, initially performing extensive research, and the development of new statistical models for incorporating pedigree, genomic, and environmental covariables (climatic and soil data). Models that incorporated genomic × environment and marker × environment and genomic × environmental covariables were earlier developed to improve the accuracy for predicting unobserved cultivars in new environments (Burgueño et al., 2012; Heslot et al., 2014; Jarquín et al., 2014; Lopez-Cruz et al., 2015; Crossa et al., 2016).

After these initial studies, an increasing number of research articles have been published effectively testing the integration of GS into conventional plant breeding pipelines for different traits measured in different environments (Crossa et al., 2017; Dreisigacker et al., 2021). The application of GS has focused on two approaches. One approach predicts the complete genetic values of individuals and focuses on both additive and non-additive effects, thereby estimating the genetic performance of candidate cultivars (Crossa et al., 2017). Additive or genetic values are predicted in breeding generations using as much phenotypic information as possible obtained from different environments in a complete or incomplete (sparse) multi-environment testing scheme (Jarquin et al., 2020). A second approach is predicting additive effects in early generations (bi-parental F2, or multi-parental populations) to achieve a rapid selection cycle with a short interval (Vivek et al., 2017; Zhang et al., 2017; Beyene et al., 2021). In these instances, the main focus is on the prediction of breeding values of the genotypes. The application of GS offers attractive benefits but comes with challenges when implemented into current conventional breeding systems.

Genomic selection is affected by a range of factors occurring at different levels. For example, one complexity arises while incorporating genotype × environment (G × E) interaction into statistical models. Also important are the genome interactions related to G × E interactions for multi-traits and the complexity of the traits (complex vs. simple) evaluated in multiple environments. Some of these complexities can be addressed using parametric models where the effect of phenotypic lines can be replaced by *g*_*j*_ expressed as a linear regression of the line phenotype on marker covariates (this approximates the genetic value of the line). The matrix ***G*** is a genomic relationship matrix with markers centered and standardized (VanRaden, 2007), which leads to what is known as Genomic Best Linear Unbiased Predictor (GBLUP). The genomic relationship matrix ***G*** is the most common parametric linear kernel that accounts for the additive relationship between lines. Also, the effect of the line can be replaced by ***A***, the additive relationship matrix of the linear kernel is derived from pedigree and proportional to the identical by descent (IBD) probabilities.

Semi-parametric genomic regression methods are efficient for capturing non-additive variation. The Reproducing Kernel Hilbert Space (RKHS) method was initially used in animal breeding (Gianola et al., 2006; Gianola and Van Kaam, 2008; Gonzalez-Recio et al., 2008) and in wheat genomic-assisted plant breeding with very promising practical results (de los Campos et al., 2009, 2010; Crossa et al., 2010; González-Camacho et al., 2012; Pérez-Rodríguez et al., 2012). Semi-parametric models use kernel methods capturing non-linear relationships between the phenotype and genotype for complex traits, such as grain yield. The Gaussian Kernel (GK) or RKHS method is a non-linear kernel (González-Camacho et al., 2012) that captures major and complex marker effects in addition to their interaction effects. Note that the non-linear kernels and the linear kernels can be employed for a single environment model and on a genomic multi-environment model, such as G × E. According to de los Campos et al. (2009); Crossa et al. (2010); Pérez-Rodríguez et al. (2012), and Cuevas et al. (2017), it is well known that the GK is efficient for capturing additive × additive epistasis interactions in multi-environment trials.

While GS is routinely deployed in the stage 1 yield trials of the CIMMYT Global Wheat Program, genomic prediction has not yet been applied in early generations due to a number of factors including, but not limited to, genetic complexity of the crop, logistics, and expense of establishing a faster cycle integrated into the existing shuttle breeding method, which involves moving seed within and/or outside Mexico each breeding generation. However, from the 2009–2010 to 2014–2015 seasons, a large GS proof-of-concept experiment was carried out with the objective of incorporating genomic prediction for increased yield in the early stages of population improvement in the context of the standard methodology applied in the CIMMYT Wheat Breeding Program in Mexico. Here, we present the results of this initial experiment, which is the first reported in wheat applying GS as early as the F2 generation. Note that the genome-based models incorporating G × E were not yet available during the time this experiment was conducted, so were not applied in this study.

## Materials and Methods

### Training and Prediction Sets

#### Composition of the Base Training Set

**FIGURE 1:**
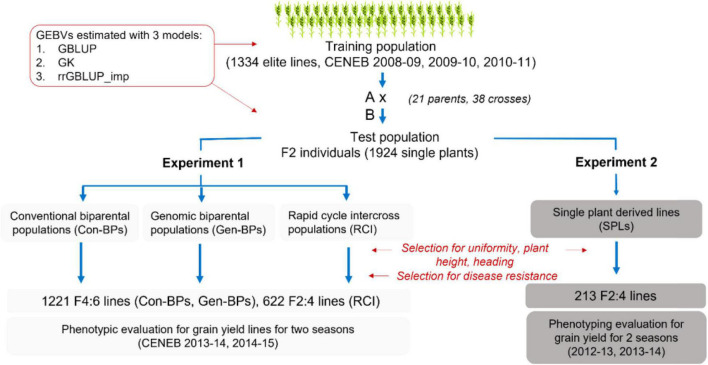
Overview of the development of populations to validate early generation genomic prediction. Con-BPs, conventional biparental populations; Gen-BPs, genomic biparental populations; GBLUP, genomic best linear unbiased prediction; rrGBLUP, Ridge Regression BLUP; SPLs, single plant-derived lines; GK, Gaussian Kernel.

The training set was comprised of 1,334 entries from the 17th and 18th Semi-Arid Wheat Yield Trials (17th and 18th SAWYT), and International Bread Wheat and Semi-Arid Wheat Screening Nurseries (29th and 30th SAWSN, 45th IBWSN; Figure 1 and Supplementary Table 1).

#### Development of Populations to Validate Early Generation Genomic Prediction

This study sought to incorporate genomic prediction for increased yield in the early stages of population development in the context of the standard breeding methodology applied at CIMMYT in Mexico. This method used selected bulks and two field generations per year alternating between the CIMMYT Experimental Station in Toluca (Lat 19° N, Long 99° W Elevation 2,640 masl) and the Campo Experimental Norman E. Borlaug (CENEB) station at Cd. Obregon (Lat 27° N, Long 110° W, Elevation 39 masl). The phenotypic selection in segregating generations was for semidwarf plant height, phenology equivalent to parents and checks and disease resistance; notably stripe rust (*Puccinia striiformis* f. sp. *tritici*), leaf rust (*Puccinia triticina*), and septoria tritici blotch (*Zymoseptoria tritici*).

Thirty-eight biparental breeding populations were generated from crosses between 21 parent lines selected from the training set and advanced to F_2_ (Supplementary Table 2). Parents were selected to limit segregation for height and phenology. From these crosses, four sets of sub-populations were derived as follows (Figure 1 and Supplementary Table 2).

#### Conventional Biparental Populations

Conventional biparental populations (Con-BPs) comprised lines derived from a random sample of approximately 1,000 F2 seeds per cross. These Con-BP F2s were each sown in a 10 m × 1.6 m plot at the CENEB station in the 2011–2012 season, and approximately 50 F2 plants with desirable height, phenology, and disease reaction were selected to form an F3 bulk. Approximately 1,000 seeds from each F3 bulk were planted in 10 m × 1.6 m plots in Toluca in May 2012, and 50 plants with desirable plant type and disease reaction were selected and harvested to form an F4 bulk. Again, 1,000 seeds of each F4 bulk were planted in the same plot configuration, 50 plants per plot were selected for plant type and disease reaction and each harvested individually to form F4:5 single plant selections. These were increased in single 2 m double-row beds over summer 2013 at the Toluca Station. We aimed to select 20 lines from each cross based on uniformity, plant type, and disease reaction. Selected rows were individually harvested and threshed to generate the F4:6 lines that were planted in field trials at the CENEB station in the 2013–2014 and 2014–2015 seasons.

#### Genomic Biparental Populations

Genomic biparental populations (Gen-BPs) were formed from 50 F2 plants per cross that were space planted at the same time and in the same field location with the Con-BP F2 subpopulations. DNA was extracted from leaf tissue of F2 individuals for genotyping-by-sequencing (GBS) and calculation of Genomic Estimated Breeding Values (GEBVs). Individuals from each cross were selected on the basis of GEBV, plant type, and disease reaction. As GEBVs from the different prediction methods were not highly correlated (see section “Results”), with no way to know which was most predictive, F2s with the highest average GEBV across the three prediction methods were selected. Selfed seed from selected F2 plants within each cross was combined to form F3 bulks. Gen-BPs were advanced from F3 bulk to F4:6 line concurrently, with the same methods and in the same field nurseries as the Con-BPs. In other words, selection methodologies and intensities were identical for Gen-BPs and Con-BPs from the F3 bulk stage. Similar numbers of lines were derived from the Gen-BP and Con-BP subpopulations of most crosses. Six crosses did not produce progeny with acceptable combinations of plant type and disease reaction for Gen-BP and Con-BP subpopulations.

#### Rapid Cycle Intercross Populations

Rapid cycle intercross populations (RCIs) were generated by crossing selected F2 individuals, those with the highest average GEBVs across prediction methods, within and between Gen-BP subpopulations. For intercrosses within a population, average GEBV and genetic distance based on the kinship matrix (VanRaden, 2008) among individuals were used to increase the probability of combining distinct, favorable alleles. All plants selected for crossing also produced enough selfed seed to contribute approximately the same number to the Gen-BP F3 bulks as plants that were not selected for intercrossing. A total of 37 RCI populations were generated. RCI F1s were space planted by cross at the Toluca Research Station in the summer of 2012 in the same field and under the same conditions as the Gen-BP and Con-BP F3 bulks. Plants were selected based on plant type and disease reaction. Selected plants were bulked by cross to form RCIF2 bulks and were then advanced concurrently with the same selection methods as for the Con-BP and Gen-BP subpopulations to produce RCI F2:4 lines for field trials at the CENEB station in the 2013–2014 and 2014–2015 seasons. The 37 RCI populations were represented by a variable number of selections although a total of 26 populations produced 16 or more selections and only 5 populations produced fewer than 10 selections. Overall, 622 lines were derived from RCI populations.

#### Single Plant-Derived Lines

Single plant-derived lines (SPLs) were developed from a subset of 240 F2 plants from across the Gen-BP subpopulations. The selection of F2 plants was based on a visual assessment of acceptable plant height, phenology, and agronomic type, without consideration of disease reaction or GEBV. F3 seed from each selected plant was sown in a single row at CIMMYTs El Batan Research Station in May of 2012. Rows were sprayed with a fungicide to control diseases and were assessed for uniformity, height, and phenology. Rows were discarded only if they expressed excessive height, slow phenological development, or high levels of within-row variability. From the 240 rows, 213 F2:4 SPLs were selected for field testing to assess response to selection for F2 GEBV using each of the three different GEBV calculation methods. SPLs were obtained from 36 of the 38 Gen-BP subpopulations and tested in field trials at CENEB in the 2012–2013 and 2013–2014 seasons.

### Field Trials and Phenotyping

#### Training Set

Phenotypic data for the training set of 1,334 lines were generated in field trials at CENEB, Cd. Obregon over the 2008–2009, 2009–2010, and 2010–2011 growing cycles under irrigated conditions with management to achieve high yield according to local best practice. Summary data for these trials are outlined in Table 1.

**TABLE 1:** Training set experiments summary data.

Trial	17SAWYT		18SAWYT		29SAWSN			30SAWSN			45IBWSN		
Season	2009	2010	2009	2010	2009	2010	2010	2010	2011	2011	2010	2011	2011
**Experiment**													
Type	Bed	Flat	Bed	Bed	Bed	Bed	Flat	Bed	Bed	Flat	Bed	Flat	Bed ZT
Entries	50	50	43	43	264	264	264	264	264	264	780	780	780
Reps	2	2	1	2	1	3	3	1	3	3	3	3	3
Mean DTH	84.7	83.6	80.5	81.5	80.1	85.1	–	84.6	91.0	87.4	88.6	91.9	95.1
Mean HT	112.0	93.3	102.3	100.0	99.1	109.8	–				104.7	113.0	100.0
Mean YLD	7.15	6.87	6.85	5.65	7.20	7.16	6.95	7.12	7.17	5.82	7.70	7.66	6.63
*H*^2^ YLD	0.67	0.45	0.58	0.70	0.63	0.83	0.81	0.64	0.72	0.78	0.88	0.89	0.68
CV YLD	7.76	11.98	7.72	7.40	6.90	9.84	12.05	8.91	7.61	12.43	8.07	10.00	10.46

*Data for the training sets included wheat lines tested in several international trails (17–18 SAWYT, 20SAWSN, 30SAWSN, and 45IDWSN) during seasons 2009, 2010, and 2011 using two planting systems bed and flat. Heritability (H^2^) and coefficient of variation (CV) of grain yield (YLD, ton/ha) and an average of days to heading (DTH, days), and height (HT, cm).*

#### Testing Set

Field trials of the developed populations were conducted at CENEB across three growing cycles (2012–2013, 2013–2014, and 2014–2015) with equivalent management to that applied to the training set. Plots were of 4.8 m^2^ (3 m × 1.6 m). Each trial was conducted in two consecutive seasons. Trials in each growing season were planted in late November or early December and harvested in early May. Data were collected for grain yield, plant height, and heading date. Details specific to the trials related to each of the following components of our research are provided in the following sections and summarized in Table 2.

**TABLE 2:** Summary field trial data for Experiments 1 and 2.

Trial	Experiment 1 Con-BP vs. Gen-BP and RCI		Experiment 2 SPL F2 GEBV validation	
Season	2013–2014	2014–2015	2012–2013	2013–2014
**Experiment**				
Type	Bed	Bed	Bed	Bed
Design	Row-column	Row-column	Row-column	Row-column
Entries	2,000	2,000	240	240
Reps	2	2	2	2
Mean DTH*^a^*	76	72.6	80.5	78.4
*H*^2^ DTH*^a^*	0.88	0.74	0.95	0.94
CV% DTH*^a^*	1.82	2.17	3.5	3.7
Mean HT*^b^*	99.6	105.1	104.2	101.3
*H*^2^ HT*^b^*	0.48	0.72	0.66	0.65
CV% HT*^b^*	5.08	3.21	4.1	4.2
Mean YLD*^c^*	5.98	4.72	7.12	5.98
*H*^2^ YLD*^c^*	0.60	0.41	0.73	0.82
CV% YLD*^c^*	9.63	13.41	9.27	6.95

*Experiment 1 compares prediction accuracy of genomic bi-parental (Gen-BP), with conventional bi-parental (Con-BP), and rapid cycling intercross population (RCI) populations in cycles 2013–2014 and 2014–2015. Experiment 2 has single plant-derived lines (SPL) F2:4 validation.*

*^a^Days to heading from sowing.*

*^b^Height in cm to tip of ear.*

*^c^Grain yield in tons per hectare.*

### Validation of Genomic Predictions for Wheat Grain Yield

#### Experiment 1 – Conventional Biparental, Genomic Biparental, and Rapid Cycle Intercross Populations

Phenotypic data for the Con-BP, Gen-BP, and RCI-derived lines (591, 630, and 622 lines, respectively) were generated in field trials at CENEB, Cd. Obregon over the 2013–2014 and 2014–2015 crop cycles (Table 3). Entries were randomly assigned to 1 of 10 different sub-experiment blocks with each sub-experiment being a two-rep row-column design. All sub-experiments included common checks. Parents of the populations were included in the experiments and assigned randomly across sub experiments. Grain yield data of the different population types were compared, and differences were determined using the least significant difference (LSD at 5% significance). The expected response to selection was derived by multiplying the narrow sense heritability by the selection differential (*H*^2^ × *S*). The latter was calculated by dividing the mean of the selected lines by the mean of the full population.

**TABLE 3:** Experiment 1: Least significant difference (LSD), mean yield comparison of different breeding populations and checks evaluated at Cd. Obregon during 2013–2014 (Year-1) and 2014–2015 (Year-2) growing seasons.

Class	*N*	Mean (ton/ha)	Tukey grouping	
YEAR-1	[LSD (0.05) = 0.1341]			
Checks	80	6.212	A	
Con-BP	1,182	6.048	B	
Gen-BP	1,260	5.990	B	C
Parents	190	5.988	B	C
RCI	1,288	5.902		C
YEAR-2	[LSD (0.05) = 0.1288]			
Checks	80	4.916	A	
Parents	190	4.800	A	B
Gen-BP	1,260	4.741	C	B
Con-BP	1,182	4.741	C	B
RCI	1,288	4.664	C	
Combined	[LSD (0.05) = 0.093]			
Checks	160	5.564	A	
Con-BP	2,364	5.394	B	
Parents	380	5.394	B	
Gen-BP	2,520	5.366	B	C
RCI	2,576	5.283		C

*Breeding populations included the genomic bi-parental (Gen-BP), conventional bi-parental (Con-BP), and rapid cycling intercross population (RCI) and parents.*

#### Experiment 2 – Validation of F2 Grain Yield Genomic Estimated Breeding Values in Single Plant-Derived F2:4 Lines

Single plant-derived lines were tested in field trials at CENEB, Cd. Obregon in the 2012–2013 and 2013–2014 crop seasons. Experiments were two replicate row-column designs. Grain yield data for the F2:4 lines were examined for correlation to GEBVs of their respective, individual F2 progenitor plant.

### Genotyping

The wheat genotypes included in the training set and F_2_ plants, indexed by their genotypic identification number (GID), were characterized using GBS following the same procedure as described in Poland et al. (2012). Briefly, genomic DNA was extracted from seedling leaf tissue using the procedure described in Dreisigacker et al. (2016). Two enzymes PstI (CTGCAG) and MspI (CCGG) were used to digest genomic DNA. Individual samples were ligated with barcoded adapters and pooled by plate into a single library. Each library was sequenced on a single lane of Illumina HiSeq2000. A total of 45,818 single nucleotide polymorphisms (SNPs) markers were initially obtained. The filtering consisted of removing markers whose minor allele frequency (MAF) was less than 5% or had more than 80% missing values. After initial filtering, 29,999 markers were available for further analysis.

### Statistical Models and Methods

#### The Base-Line Phenotype Model for the Training Populations

This part of the analysis was performed on the six field trials that included the 1,334 entries in the training set which are outlined in Table 1. Best Linear Unbiased Estimates (BLUEs) for grain yield across trials were generated using the following linear mixed model:


$$Y_{i\,j\,k\,l}=\mu +{\text{g}}_{\text{i}}+{\text{Year}}_{j}+{\text{R}}_{k\,\left(j\right)}+{\text{B}}_{l\,\left(k\,j\right)}\,{\left(\text{g}\times \,\text{L}\right)}_{i\,j}+e_{i\,j\,k\,l}$$

where *Y*_*ijkl*_ is the phenotype of wheat line *i*-th at location *j*-th in replicate *k*-th within the block *l*-th, μ is the overall mean, Year_*j*_ is the fixed effect of the year *j*-th, R_*k*(*j*)_ is the fixed effect of the *k*-th replicate within year *j*-th, B_*l*(*kj*)_ is the random effect of the incomplete block *l*-th within replicate *k*-th and year *j*-th assumed to be independently and identically normal distributed (iid) with mean zero and variance $\sigma_{b}^{2}$, g_i_ is the fixed effect of genotype *i*-th, (g × L)_*ij*_ is the fixed effect of the genotype × year interaction, and *e*_*ijkl*_ is the random error assumed to be iid normal with mean zero and variance $\sigma_{e}^{2}$. Broad sense heritability (*H*^2^) was computed on an entry-mean basis according to Bernardo (2010) as:


$$H^{2}=\frac{\sigma_{g}^{2}}{\sigma_{g}^{2}+\frac{\sigma_{g\,y}^{2}}{y}+\frac{\sigma_{e}^{2}}{y\times r}}$$

where $\sigma_{g}^{2}$ is the genotypic variance, $\sigma_{g\,y}^{2}$ is the genotype × year interaction variance, $\sigma_{e}^{2}$ is the estimated of the error variance, *y* is the number of years, and *r* is the number of replicates. Note that different trials had different numbers of testing years, 17–18 SAWYT data had trials in years 2009 and 2010, whereas the other three trials had 3 years of testing (Table 1).

#### Genomic-Enabled Prediction Models

Meuwissen et al. (2001) were the first to propose whole-genome regression methods (GS) by jointly fitting hundreds of thousands of markers with major and small effects. In the whole-genome regression methods, the number of markers (*p*) greatly exceeds the number of data-points (*n*) available; thus, implementing regression methods poses important statistical and computational challenges. However, new developments in the area of shrinkage estimation procedures allows the implementation of whole-genome regression methods.

We considered three different models: GBLUP using additive genomic relationships (VanRaden, 2008), the GK or RKHS regression (Gianola et al., 2006) which is equivalent to a GBLUP but with a non-linear kernel, and the rrGBLUP_imp where missing markers were imputed (Endelman, 2011). The GBLUP and RKHS models were fitted using routines kindly provided by de los Campos (personal communication). Nowadays, GBLUP, RKHS, and many other models can be fitted in the BGLR package (Pérez-Rodríguez and de los Campos, 2014), which is available on the CRAN website. This software was not available at the time our study was conducted.

##### The Genomic Best Linear Unbiased Prediction Model

The regression model for wheat lines (*i* = 1, 2,…, *n*) is given by:


(1) y=μ⁢1+u+ε

where **y** is the response vector of *n* phenotypic observations, μ is the overall mean, and the random vectors of the genetic values **u** and the errors ε are independent variables with $\text{u}∼\text{N}\,\left(0,\sigma_{u}^{2}\,\text{K}\right)$ and $\varepsilon ∼\text{N}\,\left(0,\sigma_{\varepsilon }^{2}\,\text{I}\right)$, respectively, where $\sigma_{u}^{2}$ is the variance of **u**, ***I*** is the identity matrix, and **K** is a symmetric semi-positive definite matrix representing the covariance of the genetic values, and ε is the vector of random errors with normal distribution and common variance, $\sigma_{\varepsilon }^{2}$. The *p* bi-allelic centered and standardized molecular markers are represented in incidence matrix **X** of order *n* × *p* such that $\text{K}=\text{G}=\frac{{\mathrm{XX}}^{{}^{\prime }}}{p}$ is a linear kernel. Model (1) is known as GBLUP (VanRaden, 2007, 2008).

Under the conditions given above, model (1) estimates the genomic relationship by means of its linear kernel **XX**′/*p*, where *p* is the number of markers. However, a nonlinear kernel, such as the GK, can also be used (Cuevas et al., 2016). The model represented by Eq. 1 is computationally very efficient and convenient when *n* >> *p* (de los Campos et al., 2012).

##### Gaussian Kernel or Reproducing Kernel Hilbert Space Regressions

In general, the parametric genomic linear regression function has a rigid structure comprising a set of assumptions, which may not be met in GS problems. Thus, departures from linearity can be addressed by semi-parametric approaches, such as the GK or RKHS regressions (Kimeldorf and Wahba, 1971; Gianola and Van Kaam, 2008; Gianola, 2013). The GK regression for semi-parametric, genomic-enabled prediction, such as kernel regression, is necessary to reduce the dimension of the parametric space and maybe able to capture complex cryptic interaction among markers (Gianola et al., 2006, 2014). Morota and Gianola (2014) pointed out that most studies carried out so far suggest that whole-genome prediction coupled with combinations of kernels may capture non-additive variation (Gianola et al., 2014).

The basic idea underlying the GK approach to GS (Kimeldorf and Wahba, 1971; Gianola, 2013) is to use the matrix of markers **X** to build a covariance structure among genetic values **u**. Therefore, $\text{u}∼N\,\left(0,\sigma_{g}^{2}\,{\text{K}}_{h}\right)$ is independent of ε (Crossa et al., 2010; de los Campos et al., 2010), **K**_*h*_ is a symmetric positive semi-definite matrix of order *n* × *n*, known as the reproducing kernel (RK) matrix, which depends on the markers and the bandwidth parameter *h* > 0, $\sigma_{g}^{2}$> 0, and ε is an *n* × 1 vector of homoscedastic and independent normal errors.

This general approach requires choosing an RK, for example, a GK function


(2) $$K_{h}\,\left({\text{x}}_{i},{\text{x}}_{j}\right)=\exp\left(-h\,d_{i\,j}^{2}/q_{0.05}\right),$$

where **x**_*i*_ and **x**_*j*_ are the marker vectors for the *i*-th and *j*-th individuals, and *q*_*0.05*_ is the fifth percentile of the squared Euclidean distance $d_{i\,j}^{2}$ (González-Camacho et al., 2012).

##### Ridge Regression Best Linear Unbiased Prediction With Imputed Marker Data

The marker-based, additive relationship matrix was calculated with the function A.mat in R package rrGBLUP, version 4.1 (Endelman, 2011), which centers (but does not standardize) each marker by the population mean (VanRaden, 2008). The relationship matrix was additionally calculated with the imputed markers. Missing data were imputed with the “EM” option in A.mat, which implements a multivariate normal expectation-maximization (EM) algorithm (Poland and Rife, 2012).

#### A Fivefold Cross-Validation

A fivefold cross-validation was performed to evaluate the prediction performance of the models on the training set. The full dataset was randomly divided into five mutually exclusive subsets, four of which formed the training set for fitting the model, and the fifth was used as a test set. Predictive abilities were calculated as the Pearson’s correlation coefficient between the predicted values and the observed phenotypic values of the test set.

## Results

### Validation of Genomic Prediction Models

**FIGURE 2:**
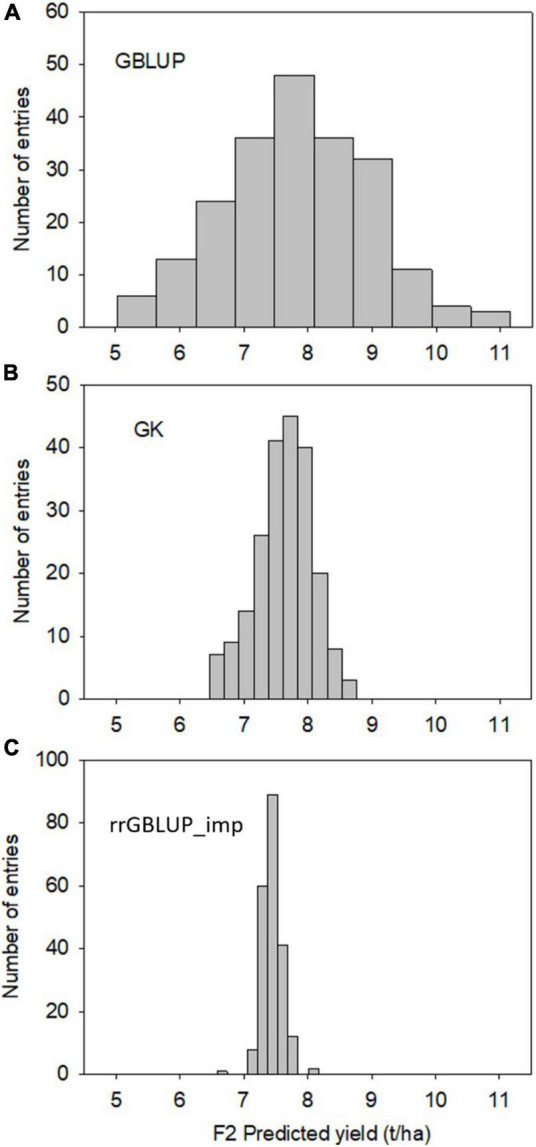
Experiment 2. Distribution of F2-predicted breeding values (grain yield) of 213 randomly selected individuals estimated using GK (top, **A**), GBLUP (Middle, **B**), and rrGBLUP (low, **C**) with imputed markers.

Predictions with GBLUP, rrGBLUP_imp, and GK in the training population had similar levels of predictive ability for a yield of 0.42–0.43 as determined by a fivefold cross-validation (Table 4). The GEBVs produced by the three methods showed high correlations of between 0.93 and 0.97 in the training set. In contrast, the models produced divergent predictions in F2 populations. The shapes of the distribution of GEBVs from each prediction method also differed with GBLUP having a wide distribution from 5 to 11 ton/ha while rrGBLUP_imp values were more narrowly grouped between 6.5 and 8.1 ton/ha (Figures 2A–C). The lack of correlation and different distributions of values caused uncertainty about which was the most appropriate method to use in the selection of F2 plants to generate genomically selected bulks and to intercross in a rapid cycle intercross strategy. As the GEBVs were uncorrelated, not negatively correlated, individuals with the highest GEBVs averaged across the prediction methods were selected for selfing to form F3 bulks and for intercrossing to form the RCI populations. Phenotypic selection was also applied for plant height, phenology, and disease reaction in the same way as for the Con-BP populations. For the RCI populations, the additional criteria of maximizing genetic differences between F2 individuals, if selected from the same biparental cross, were applied in planning intercrosses.

**TABLE 4:** Predictive power of GEBVs in a training population of 1,334 inbreds and correlation between GEBV predictions for grain yield by three different calculation methods (GBLUP, rrGBLUP_imp, and GK) among inbreds in the training set and in a target population of 1,924 F2s.

Prediction model	Yield (fivefold cross validation)	GBLUP	rrGBLUP_imp
		Training/F2	Training/F2
GBLUP	0.43	–	–
rrGBLUP_imp	0.42	0.96/0.44	–
GK	0.42	0.97/0.37	0.93/0.13

*GBLUP, genomic best linear unbiased prediction; rrGBLUP, Ridge Regression BLUP; GK, Gaussian Kernel.*

Because populations were advanced through a selected bulk method to develop the material tested in Experiment 1, this experiment could not address the question of whether one method was superior to another in F2 GEBV calculation. Therefore, a random subsample of F2 plants was chosen to develop single F2 plant-derived lines so a correlation between the yield of a derived line and GEBV of an F2 could be measured. This set of lines was the basis for Experiment 2.

### Experiment 1 – Conventional Biparental, Genomic Biparental, and Rapid Cycle Intercross Populations

A total of 1,857 lines were derived from conventional, phenotypically selected biparental (Con-BP), Gen-BP, and RCI breeding methods with roughly similar numbers from each (Supplementary Table 2). All methods used phenotypic selection to progress material through selected bulk stages, while the Gen-BP method used GEBVs of F2 plants to add a single cycle of GS and RCI used the F2 GEBVs to select plants for intercrossing to produce new populations that were subsequently passed through the same phenotypic selection methodologies.

**FIGURE 3:**
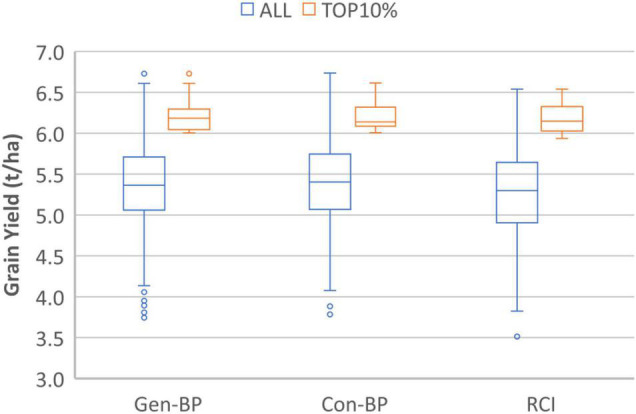
Experiment 1. Box-plots comparing grain yield distribution of all the lines for Genomic Bi-parental (Gen-BP), Conventional Bi-parental (Con-BP), and Rapid Cycle Intercross population (RCI) populations (blue box-plots) and their corresponding top 10% entries (red box-plots).

**FIGURE 4:**
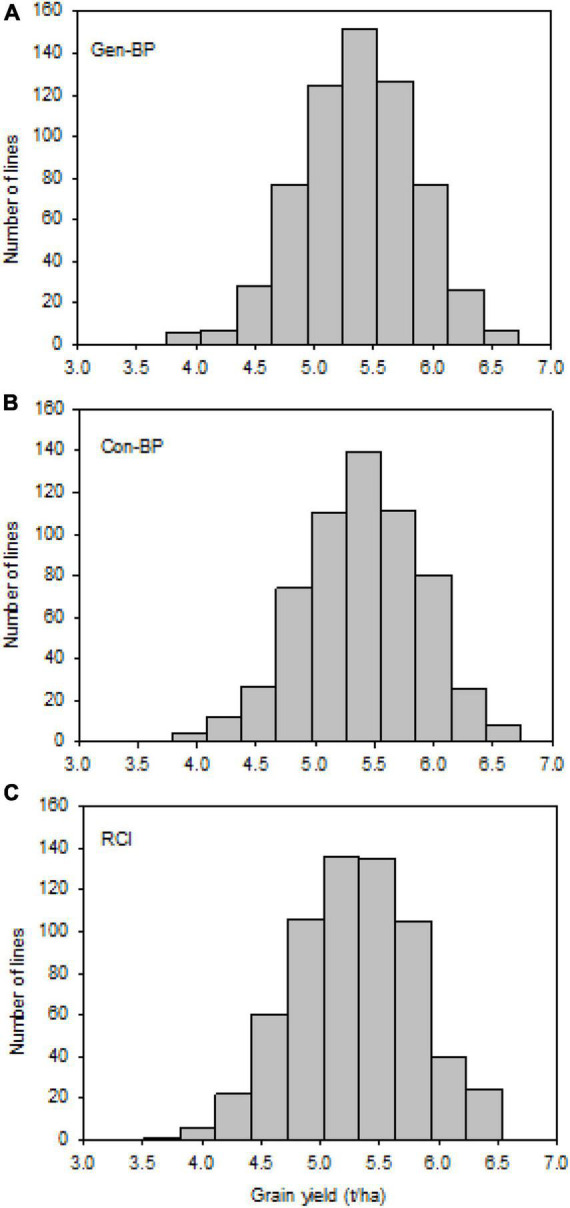
Experiment 1: Distribution of observed grain yield across 2 years under different selection strategies: **(A)** Gen-BP, **(B)** Con-BP, and **(C)** RCI. Con-BPs, conventional biparental populations; Gen-BPs, genomic biparental populations; RCI, rapid cycling intercross.

Field testing showed that Con-BP lines yielded an average of 2% more than RCI lines (*P* < 0.001), Gen-BP lines yielded an average of 1.5% more than RCI lines (*P* < 0.01), and there was no significant difference between Gen-BP and Con-BP (Table 3) populations. Similar comparisons of yield focusing on the top 10% highest yielding lines in each population type showed similar patterns with Con-BP having the highest yield, significantly greater than GS-BP and RCI (Figure 3 and Table 5). Although differences were statistically significant, they were only approximately 1%. Gen-BP subpopulations in the top 10% were not significantly different in yield to the top 10% of RCI lines. Response to selection in the RCI populations was marginally greater than for Con-BP and Gen-BP, but the difference was small and likely reflects the lower mean yield and distribution of grain yield in the RCI populations compared to the other population types (Table 3 and Figure 4).

**TABLE 5:** Experiment 1: Comparing the expected response to selection under 5 and 10% selection intensity for different selection schemes for average grain yield (AV_YLD ton/ha) derived from trials performed at the CENEB station in seasons 2013–2014 and 2014–2015.

			TOP5%			TOP10%		
Class	*N*	AV_YLD	AV_YLD	*S*	*R*	AV_YLD	*S*	*R*
Gen-BP	630	5.366	6.326	0.960	0.576	6.189	0.823	0.494
Con-BP	591	5.394	6.347	0.953	0.572	6.216	0.822	0.493
RCI	635	5.286	6.312	1.026	0.615	6.177	0.891	0.534

*Con-BPs, conventional biparental populations; Gen-BPs, genomic biparental populations; RCI, rapid cycling intercross.*

*S = Selection differential (selected mean − population mean).*

*R = Expected response to selection = H^2^ × S.*

*Expected selection response (H^2^ = 0.6).*

### Experiment 2 – Validation of F2 Grain Yield Genomic Estimated Breeding Values in Single Plant-Derived F2:4 Lines

**FIGURE 5:**
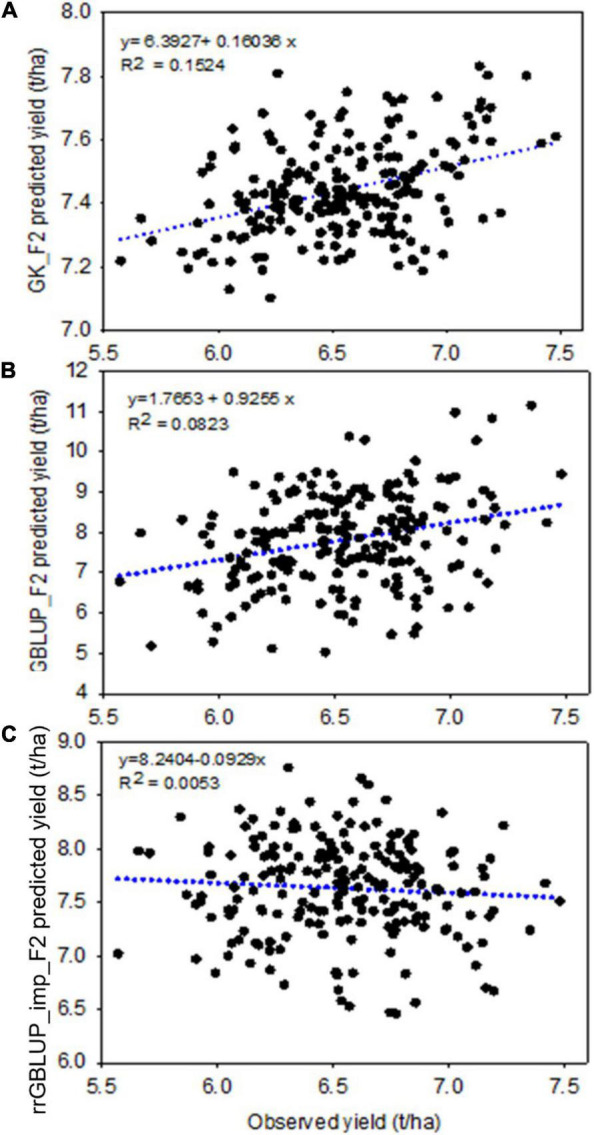
Experiment 2: Scatter plot showing the correlation between F2 predicted yield, estimated with different prediction models GK (top, **A**), GBLUP (middle, **B**), and rrGBLUP_imp (low, **C**), with an observed yield of 213 derived F2:4 lines. GBLUP, genomic best linear unbiased prediction; rrGBLUP, Ridge Regression BLUP; GK, Gaussian Kernel.

In Experiment 2, we compared the predictive ability of the different GEBV calculation methods in F2 in a set of 213 single plant-derived F2:4 lines from randomly selected F2 plants. Trials of the F2:4 SPLs showed a significant positive correlation with F2 GEBVs from GK and GBLUP (Table 6 and Figure 5). Individuals with the highest 10 and 20% GEBVs predicted by GK, produced F2:4 progeny lines with realized grain yield gains of 4.7 and 4.2%, respectively; significantly higher than the mean of 50 random samples from across the full set of F2s (Table 7). The top 10 and 20% of F2s predicted by the GBLUP method showed realized gains of 3.68 and 2.60%, respectively, in their F2:4 progenies; significantly higher than the mean of 50 random samples of the same proportions (Table 7). Contrarily, selecting the top 10 and 20% of F2 GEBVs estimated with rrGBLUP_imp did not produce F2:4 progenies with a higher mean performance compared to random samples.

**TABLE 6:** Experiment 2: Correlations between F2:4 GEBVs from three prediction methods (GBLUP, GK, and rrGBLUP_imp) from grain yield of 213 derived F2:4 lines across two seasons.

	F2:4 observed yield	
F2:4 predicted YLD	Correlation	*P*-Value
GBLUP	0.2870	0.000024
GK	0.3020	0.000009
rrGBLUP_imp	−0.0733	0.290000

*GBLUP, genomic best linear unbiased prediction; rrGBLUP, Ridge Regression BLUP.*

**TABLE 7:** Yield of F2:4 lines based on selection of the top 10 and 20% of GEBVs from different methods (GBLUP, GK, and rrGBLUP_imp) compared to a random sample of 10 and 20% of all F2:4 lines, with 50× sampling (the top 10 and 20% *t*-test: Two-Sample Assuming Unequal Variances.

	Sample	Best 20% F2:4-predicted lines			Sample	Best 10% F2:4-predicted lines		
	20%	GK	GBLUP	rrGBLUP_imp	10%	GK	GBLUP	rrGBLUP_imp
Mean yield (ton/ha)	6.52	6.76	6.69	6.49	6.52	6.83	6.80	6.47
Variance	0.0036	0.1536	0.1081	0.0808	0.0077	0.1107	0.1334	0.0989
Observations (*n*)	50	42	42	43	50	21	20	21
Mean difference (*D*)		0.24	0.17	−0.03		0.31	0.28	−0.05
% Mean difference		3.68	2.61	−0.46		4.75	4.29	−0.77
Degree of freedom		43	43	45		21	20	21
*t*-Value		3.9823	3.3382	−0.6783		4.2767	3.3561	−0.7761
*P* (*t* < = *t*) one-tail		0.0001	0.0009	0.2505		0.0002	0.0016	0.2232
*t* critical one-tail		1.6811	1.6811	1.6794		1.7207	1.7247	1.7207
*P* (*t* < = *t*) two-tail		0.0003	0.0017	0.5011		0.0003	0.0031	0.4463
*t* critical two-tail		2.0167	2.0167	2.0141		2.0796	2.086	2.0796

*GBLUP, genomic best linear unbiased prediction; rrGBLUP, Ridge Regression BLUP; GK, Gaussian Kernel.*

**FIGURE 6:**
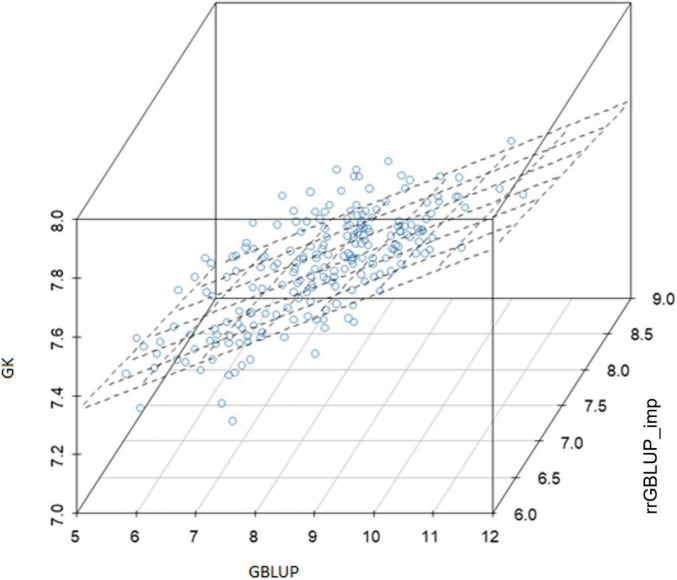
Experiment 2: Relationship between genomic-enabled predictive values of 213 F2 which were later advanced to F4 (F2:4) using models GK, GBLUP, and rrGBLUP_imp. GBLUP, genomic best linear unbiased prediction; rrGBLUP, Ridge Regression BLUP; GK, Gaussian Kernel.

Within the subset of 213 F2s which were randomly sampled to produce F2:4 bulks for yield testing, the correlations between prediction methods were also low (Table 4 and Figure 6). Figures 5A–C shows scatterplots of F2 single plant GEBVs vs. realized yields of derived F2:4 lines (GEBVs on *Y*-axis, yields on *X*-axis). From these, it is clear that correlations between F2 GEBVs and F2:4 yield for GK and GBLUP are not strong but are not driven by outliers with high leverage. In both cases, the selection of F2s with the highest GEBVs would avoid the selection of the lowest yielding F2:4 lines.

## Discussion

The proof-of-concept Experiment 2 reported here demonstrates the potential of early generation genomic prediction to increase genetic gain over conventional selection methods by allowing the ability to increase the number of crossing cycles per year. In Experiment 2 of our study, F2 GEBVs generated by GK and GBLUP methods showed significant positive correlations with the yield of derived lines. The highest 10 and 20% of GEBVs from the GK method showed 4.7 and 4.2% increases, respectively, and the top 10 and 20% of F2s GEBVs predicted by GBLUP showed realized gains of 3.68 and 2.60% over a 50× random sample of the same proportion of lines from the same populations. In contrast, a similar analysis of F2 GEBVs from the rrGBLUP_imp method showed no difference from the mean of the 50× random sampling.

Each of the three prediction methods used in this study produced highly correlated GEBVs in inbreds and the same levels of predictability of inbred performance based on cross validation in a training set of elite CIMMYT inbreds. In contrast, predictions in F2s derived from crosses between inbreds that were part of the training set for the model showed little to no correlation and differing levels of predictive ability compared with a realized yield of F2 SPLs. These differences are likely due to the different abilities of the prediction methods to handle heterozygosity, which is generally not accurately characterized with a GBS genotyping platform and the importance of non-additive variation in wheat. This may be reflected in the much narrower distribution of GEBVs from rrGBLUP_imp compared to GBLUP and particularly GK. The difference in the distribution of the GEBVs between the GBLUP and GK methods is likely due to the different shrinkage applied in each method. On the other hand, differences between GBLUP and rrGBLUP_imp are likely due to the imputation method used.

In Experiment 1, we attempted to incorporate F2 genomic prediction into a selected bulk breeding methodology closely mirroring the typical breeding methodology in the CIMMYT spring bread wheat program. The three different prediction methods generated F2 GEBVs that showed little correlation with one another. It should be noted that the low correlations between the rrGBLUP_imp with GBLUP and GK were considered as a rare result, especially knowing the equivalence between the GBLUP and the rrGBLUP_imp. The reasons for the failure of the rrGBLUP_imp in generating similar predictions to GBLUP are unknown but may be attributable to different factors. For example the nature of the imputation algorithm or convergence issues with the Expectation-Maximization algorithm in rrGBLUP_imp. Since the three methods had similar ability to predict yield of inbreds and predictions were correlated, it was difficult to discard one of the models based on observed phenotypes and we decided to use an average of the methods. If we had conducted additional research to confirm that GK was the most predictive method or that GBLUP also showed a useful level of predictability, we would likely have made better selections of F2 individuals to form selected bulks and to make early generation intercrosses. Given that our selections were probably no better than random and the number of F2s selected was less than in parallel conventionally selected populations, it is hardly surprising that a lower level of genetic variance (presumed by planting only 5% of the number of F2s in Gen-BP vs. Con-BP) did not result in a yield advantage in the genomically selected biparental-derived inbreds (Gen-BP) and the early generation intercross derived (RCI) inbred populations; both on average and in the highest yielding 20% of lines from each of the population types.

When comparing genome-based predictions, we should also emphasize that in this study the accuracy of the three methods (GBLUP, GK, and rrGBLUP_imp) for predicting F2 plants was measured at the F2:4. Therefore, any attempt to make a precise estimate of errors among the three methods and benchmarking results from genome-based methods with those under conventional breeding methods in terms of biases and errors are complex and out of the scope of this research. It would be worthwhile to investigate further methods to optimize prediction power in early generation wheat populations. If a robust method can be determined, there are useful increases in genetic gain from early generation genomic prediction in wheat, particularly, in populations that are not varying for some of the obvious drivers of yield that are easily selected phenotypically, such as height or flowering time. Considering there are roughly one million F2 plants generated per year in the CIMMYT spring bread wheat program, early generation genomic prediction will likely be best targeted to certain types of populations that provide the greatest probability of higher response to selection or where there is little obvious variation amenable to phenotypic selection.

These evaluations give the first indications of genetic gains from early generation GS for a highly complex trait in a practical wheat breeding program.

## Data Availability Statement

The original contributions presented in the study are publicly available. This data can be found here: https://data.cimmyt.org/dataset.xhtml?persistentId=hdl:11529/10548576.

## Author Contributions

All authors listed have made a substantial, direct, and intellectual contribution to the work, and approved it for publication.

## Conflict of Interest

The authors declare that the research was conducted in the absence of any commercial or financial relationships that could be construed as a potential conflict of interest.

## Publisher’s Note

All claims expressed in this article are solely those of the authors and do not necessarily represent those of their affiliated organizations, or those of the publisher, the editors and the reviewers. Any product that may be evaluated in this article, or claim that may be made by its manufacturer, is not guaranteed or endorsed by the publisher.
