# Supplementary material for: Response to Early Generation Genomic Selection for Yield in Wheat
Source: Front Plant Sci. 2022 Jan 11;12:718611. doi: 10.3389/fpls.2021.718611 (PMC8787636; doi:10.3389/fpls.2021.718611)
Supplement: Supplementary file 1 [file Table_1.docx]

**Supplemental Table S1**- Parent lines used in crosses to develop experimental populations

| **Parent No.** | **GID** | **Pedigree** |
| --- | --- | --- |
| 1 | 5894654 | SOKOLL//SUNCO/2*PASTOR |
| 2 | 5894994 | MTRWA92.161/PRINIA/5/SERI*3//RL6010/4*YR/3/PASTOR/4/BAV92 |
| 3 | 5895340 | SOKOLL/92.001E7.32.5 |
| 4 | 5999777 | BABAX/LR42//BABAX/3/VORB |
| 5 | 6000617 | CNO79//PF70354/MUS/3/PASTOR/4/BAV92/5/FRET2/KUKUNA//FRET2/6/MILAN/KAUZ//PRINIA/3/BAV92 |
| 6 | 6000906 | SOKOLL*2/TROST |
| 7 | 6000922 | SOKOLL//PBW343*2/KUKUNA/3/ATTILA/PASTOR |
| 8 | 6001014 | GK ARON/AG SECO 7846//2180/4/2*MILAN/KAUZ//PRINIA/3/BAV92 |
| 9 | 6001235 | BAV92/SERI |
| 10 | 6001669 | BOW/VEE/5/ND/VG9144//KAL/BB/3/YACO/4/CHIL/6/CASKOR/3/CROC_1/AE.SQUARROSA (224)//OPATA/7/PASTOR//MILAN/KAUZ/3/BAV92 |
| 11 | 5423688 | TC870344/GUI//TEMPORALERA M 87/AGR/3/2*WBLL1 |
| 12 | 5429403 | PASTOR//HXL7573/2*BAU/3/WBLL1 |
| 13 | 5435731 | SOKOLL/3/PASTOR//HXL7573/2*BAU |
| 14 | 5435924 | W15.92/4/PASTOR//HXL7573/2*BAU/3/WBLL1 |
| 15 | 5436044 | MEX94.27.1.20/3/SOKOLL//ATTILA/3*BCN |
| 16 | 5686616 | CHRZ//BOW/CROW/3/WBLL1/4/CROC_1/AE.SQUARROSA (213)//PGO |
| 17 | 5894989 | MTRWA92.161/PRINIA/5/SERI*3//RL6010/4*YR/3/PASTOR/4/BAV92 |
| 18 | 5999970 | POTCH 93/4/MILAN/KAUZ//PRINIA/3/BAV92/5/MILAN/KAUZ//PRINIA/3/BAV92 |
| 19 | 6000921 | SOKOLL//PBW343*2/KUKUNA/3/ATTILA/PASTOR |
| 20 | 6001675 | BOW/VEE/5/ND/VG9144//KAL/BB/3/YACO/4/CHIL/6/CASKOR/3/CROC_1/AE.SQUARROSA (224)//OPATA/7/PASTOR//MILAN/KAUZ/3/BAV92 |
| 21 | 5423328 | BABAX/LR42//BABAX/3/ER2000 |
