# Supplementary material for: Response to Early Generation Genomic Selection for Yield in Wheat
Source: Front Plant Sci. 2022 Jan 11;12:718611. doi: 10.3389/fpls.2021.718611 (PMC8787636; doi:10.3389/fpls.2021.718611)
Supplement: Supplementary file 2 [file Table_2.docx]

**Supplemental Table S2**- Overview of populations from which lines were selected for Experiments 1 and 2.

|  |  | No. Lines | |  |  |  | No. Lines |
| --- | --- | --- | --- | --- | --- | --- | --- |
| Cross No | Pedigree^a^ | Conventional^b^ | Genomic^b^ | Single Plant^c^ | Cross No | Pedigree^a^ | RCI^b^ |
| 01 | 12/1 | 20 | 20 | 0 | 33 | 14/4//14/4 | 20 |
| 02 | 13/18 | 20 | 20 | 5 | 34 | 14/4//14/4 | 20 |
| 03 | 14/19 | 20 | 20 | 3 | 35 | 14/4//7/9 | 7 |
| 04 | 1/8 | 20 | 20 | 2 | 36 | 6/8//6/8 | 20 |
| 05 | 3/7 | 20 | 20 | 3 | 37 | 6/8//6/8 | 20 |
| 06 | 12/5 | 11 | 27 | 1 | 39 | 11/19//11/19 | 20 |
| 07 | 13/16 | 20 | 20 | 2 | 40 | 6/7//6/7 | 20 |
| 08 | 14/5 | 18 | 21 | 2 | 41 | 6/7//6/7 | 20 |
| 09 | 1/5 | 20 | 20 | 3 | 42 | 7/9//5/8 | 20 |
| 10 | 2/8 | 20 | 19 | 6 | 43 | 7/9//6/7 | 20 |
| 11 | 221/18 | 20 | 20 | 4 | 44 | 6/7//4/5 | 20 |
| 12 | 15/16 | 20 | 20 | 3 | 45 | 6/7//5/8 | 20 |
| 13 | 1/3 | 17 | 20 | 0 | 46 | 6/7//5/8 | 20 |
| 14 | 2/9 | 20 | 20 | 6 | 47 | 6/7//5/8 | 17 |
| 15 | 11/19 | 20 | 20 | 3 | 48 | 6/7//5/8 | 19 |
| 16 | 12/8 | 18 | 18 | 4 | 49 | 6/8//4/10 | 20 |
| 17 | 1/10 | 5 | 12 | 4 | 50 | 6/8//2/9 | 20 |
| 18 | 3/8 | 20 | 20 | 5 | 51 | 6/7//4/10 | 21 |
| 19 | 14/4 | 20 | 18 | 11 | 52 | 6/7//4/10 | 13 |
| 20 | 7/9 | 20 | 20 | 4 | 53 | 4/5//4/5 | 12 |
| 21 | 6/7 | 22 | 18 | 20 | 54 | 5/8//5/8 | 20 |
| 22 | 6/8 | 22 | 18 | 12 | 55 | 5/8//5/8 | 18 |
| 23 | 3/6 | 20 | 20 | 6 | 56 | 3/6//3/6 | 6 |
| 24 | 5/8 | 18 | 22 | 13 | 57 | 4/10//4/10 | 20 |
| 25 | 3/4 | 17 | 21 | 2 | 58 | 2/7//3/6 | 12 |
| 26 | 4/5 | 18 | 17 | 3 | 59 | 2/7//2/7 | 20 |
| 27 | 4/10 | 6 | 20 | 6 | 60 | 2/7//2/7 | 7 |
| 28 | 2/5 | 20 | 18 | 11 | 61 | 3/4//3/4 | 13 |
| 29 | 2/6 | 20 | 20 | 22 | 61 | 3/4//3/4 | 20 |
| 30 | 2/7 | 20 | 20 | 10 | 62 | 2/5//2/5 | 16 |
| 31 | 1/6 | 20 | 20 | 7 | 63 | 2/6//2/6 | 20 |
| 32 | 2/4 | 20 | 20 | 9 | 64 | 2/6//2/6 | 2 |
| 33 | 7/8 | 0 | 0 | 4 | 65 | 2/6//2/6 | 8 |
| 34 | 8/9 | 0 | 0 | 2 | 66 | 2/4//1/6 | 11 |
| 35 | 13/10 | 0 | 0 | 2 | 67 | 2/4//1/6 | 20 |
| 36 | 7/10 | 0 | 0 | 7 | 68 | 2/4//2/4 | 20 |
| 37 | 15/20 | 0 | 0 | 1 | 69 | 2/4//2/4 | 20 |
| 38 | 3/10 | 0 | 0 | 5 |  |  |  |
| Total Lines |  | 591 | 630 | 213 |  |  | 622 |

^a^Purdy style abbreviated pedigree based on parental line number as outlined in **Supplemental Table S1**

^b^Lines tested in Experiment 1

^c^Lines tested in Experiment 2
